# Cultural awareness workshops: limitations and practical consequences

**DOI:** 10.1186/s12909-018-1450-5

**Published:** 2019-01-08

**Authors:** Stephane M. Shepherd

**Affiliations:** 1 Centre for Forensic Behavioural Science, Swinburne University of Technology, Alphington, Australia; 2 Bloomberg School of Public Health, The Johns Hopkins University, Baltimore, MD USA

**Keywords:** Cultural awareness, Cultural competence, Cultural humility, Cultural safety, Cultural sensitivity, Cultural intelligence, Health professionals, Cross-cultural health care, Cross-cultural psychology

## Abstract

Cultural awareness training for health professionals is now commonplace across a variety of sectors. Its popularity has spawned several alternatives (i.e., cultural competence, cultural safety, cultural humility, cultural intelligence) and overlapping derivatives (diversity training, anti-racism training, micro-aggression training). The ever-increasing reach of cultural awareness initiatives in health settings has generally been well intentioned - to improve cross-cultural clinical encounters and patient outcomes with the broader expectation of reducing health disparities. Yet the capacity of cultural awareness training to accomplish or even impact such outcomes is seldom comprehensively scrutinized. In response, this paper applies a much needed critical lens to cultural awareness training and its derivatives by examining their underpinning philosophies, assumptions and most importantly, verification of their effectiveness. The paper finds cultural awareness approaches to be over-generalizing, simplistic and impractical. They may even induce unintended negative consequences. Decades of research point to their failure to realize meaningful outcomes in health care settings and beyond. Broader expectations of their capacity to reduce health disparities are almost certainly unachievable. Alternative suggestions for improving cross-cultural health care interactions and research are discussed within.

## Background

Cross-cultural training and education (also known as cultural awareness training) is commonly offered to professionals, students and volunteers across numerous industries for the purposes of enhancing effective cross-cultural interaction. Its origins can be traced back to early diversity training efforts in the United States following civil rights legislation in the 1960s [1]. Soon after, public health administrators sought to address the mechanisms underpinning reported cross-cultural health disparities [2]. Bias and/or cultural differences between health care service providers and migrant/minority populations are believed to contribute to both the poor health care experiences and subsequent unmet health needs of underprivileged minority groups [3–9]. As such, learning about the worldviews, norms and practices of cultural minority clientele is anticipated to not only endow health care professionals with a better capacity to understand and serve their patients, but to also reduce broader health care inequities in the process [8, 10–13]. The cultural awareness industry has now become a global phenomenon and commercial behemoth, grossing billions of dollars annually. Cultural awareness training is commonplace and often mandated in multiple sectors. Its philosophies are embedded in the strategic plans and mission statements of organisations and branches of government. Human resource departments ensure that employees abide by such protocols. Cultural awareness standards and trainings are often components of university course curricula. There are now multiple off-shoots (i.e., cultural competence, cultural safety, cultural humility, cultural intelligence) and overlapping derivatives (diversity training, anti-racism training, micro-aggression training). Some concepts (i.e., cultural competence) have spawned their own academic disciplines, with their own experts, theories and bodies of literature [14].

The ever-increasing reach of cultural awareness endeavours has generally been well intentioned. Improving inter-cultural dealings in a paradigm of globalism and growing diversity seems eminently sensible. Perhaps even more so in health settings where clinical encounters can impact patient actions [15–19]. Yet the utility of cultural awareness education has rarely been methodically unpacked and scrutinized. A cursory search for academic literature on cultural awareness training will recover hundreds of thousands of papers on the concept(s) and their presumed advantages. In contrast, fewer than two dozen articles offer robust appraisals or critiques of cultural awareness, with half of these proposing that existing trainings should in fact be more trenchant and/or expansive. This ostensible unevenness in the literature warrants some re-visiting. Is this seemingly near-unanimity in the literature a judicious reflection of a robust evidence base? In response, this commentary applies a much needed critical lens to cultural awareness training and its derivatives by examining its philosophies, assumptions and most importantly, verification for its effectiveness. The critiques offered in this paper are partially derived from clinical experiences, and the author’s long time professional involvement in the development, oversight and presentation of cross-cultural trainings. The author has also attended over a dozen separate cross-cultural education workshops across two regions (North American and Oceania). Greater consideration in the paper is afforded to the implications for clinical encounters given that cultural awareness initiatives have conventionally been oriented to health care settings.

## Main text

### The workshop

The flagship component of cultural awareness education is the workshop. These vary in duration ranging from a single session of 1–2 h to full-days held across multiple sessions. The method and intensity of instruction also vary including the array of activities available to attendees. Though cosmetically variable, workshop content which is didactic in nature, encompasses at least one of five overlapping themes – historical matters, belief systems, (clinical) interaction approaches, discrimination and organisational issues. *Historical matters* provide a historical backdrop germane to whichever cultural group is the focus of the workshop. Injustices are often emphasized here – colonialism, discriminatory legislation, forced acculturation, land dispossession, oppression. For recent culturally diverse migrant groups, pre and post migratory stressors and re-settlement challenges will be articulated. The rationale is to connect unjust historical episodes and precarious migration experiences to contemporary social circumstances, disadvantages and behaviours. The second theme, *belief systems*, refers to cultural differences in worldview, norms and practices. A cultural group’s family structures, social hierarchies and religious/spiritual conventions are outlined with regard to how they shape community/familial expectations and responsibilities. Here, the collectivist orientation of particular cultural groups is often posited to be at odds with Western notions of individualism. This is extended to conceptualizations of health and mental health where it is often emphasized that biomedical approaches to medicine are embedded in frameworks that epitomize Western values and assumptions. The biomedical model is described as narrowly focused on direct causes and solutions for illness and perceived to be in contrast with holistic models of health that encompass broader notions of personal, community and meta-physical wellbeing. Workshop attendees are to be cognizant of differing explanatory models of health, social meanings of sickness and traditional remedies that may require accommodation in conventional health care settings. Clinicians may also be trained to identify culturally-unique symptom reporting styles and non-verbal cues to allow for a better reading of a client’s presentation and perhaps reduce the scope for clinical misinterpretation. The third workshop theme is focused on *interaction approaches*. In health settings, these are essentially guidelines for effective patient-provider communication. Workshop attendees may learn appropriate styles of greeting, an awareness of unique cultural taboos and stigmas, and strategies for rapport development and gaining trust. The roles of interpreters and translators are often described here. The fourth theme, *discrimination* is a key feature and driver of the contemporary cultural awareness workshop. A cultural group’s experiences of racism in workplace settings and society at large will be outlined. It is now common for workshops to talk about structural oppression and how dominant norms are entrenched into a profession or an organization’s structure, perpetuating discrimination unintentionally or otherwise. This is often discussed in tandem with power imbalances and privilege. Some workshops will discuss implicit bias and micro-aggressive language and the psychological consequences for cultural minorities. Links between racism and poor health are increasingly made known. A purported aim is to manage clients without demeaning, invalidating or disempowering their cultural identity. A solution commonly offered to address biases and power imbalances is self-reflexivity which requires an ongoing self-interrogation of one’s own prejudices. The final workshop theme refers to *organisational/institutional issues*. These are essentially management policies that are implemented to support cultural awareness principles at a systemic level. Initiatives may include engagement/consultation with local multi-cultural communities, hiring strategies to diversify the workforce, staff mentoring, health promotion for underserved groups and the implementation of anti-discrimination procedures. The five workshop themes presented, though not an exhaustive list, are typically canvassed to some extent in the course of cultural awareness education.

### The philosophies

The content of a workshop will depend on its underpinning philosophy. Over the past four decades, several popular cross-cultural communication concepts have emerged. These include, but are not limited to cultural awareness, cultural competence, cultural safety, cultural humility and more recently, cultural intelligence. The concepts overlap yet vary in scope and possess differing foci or ‘starting points’. The objectives of each concept and their respective alignment with the five workshop themes above, will be briefly examined. The term ‘cultural awareness’ is often loosely employed to describe all forms of cross-cultural education (as in this article), however it also refers to a particular style of training. *Cultural awareness* was the first structured program of cultural education, originating in the USA in the 1960s and its iterations are still the most common form of training. Although touching on all five workshop themes, cultural awareness has a specific focus on belief systems (cultural norms and traditions) with some attention on interaction approaches and historical issues [20]. Training is geared towards expanding the cross-cultural knowledge of the individual. *Cultural competence* was developed in the USA in the 1980s [5] to foster improved care for minority children with mental health concerns. It is a more systemic approach focusing on both personal attitudes, communication and organisational policies [4]. Workshop themes 4 (Discrimination) and 5 (Organisational issues) are emphasized. Training at the personal level borrows heavily from cultural awareness however individuals are also encouraged to engage in an ongoing process of self-reflexivity. Strategies to expand an organisation’s capacity to support and implement culturally competent protocols are underscored. *Cultural safety* was pioneered by Maori nurses in New Zealand in the 1990s [21]. The focus is primarily on workshop theme 4 (Discrimination) though some consideration is afforded to the other four themes. Cultural safety implores professionals to not just interrogate their own cultural belief systems through self-reflexivity, but to acknowledge how the vocation itself may have built-in or entrenched dominant culture norms and standards that serve to maintain power imbalances and structures of oppression which play out in health service delivery (particularly for Indigenous patients). *Cultural humility* is another US-developed concept originating in the 1990s [22]. Inspiration for cultural humility derived from recognizing the limitations of aspects of cultural awareness and cultural competence. Mastering a client’s cultural background was deemed to be unachievable and a commitment to contending with one’s own biases (workshop theme 4), favoured. There is considerable overlap with cultural safety, although less effort is expended on learning about the ‘other’ with more emphasis on empowering the client to determine if and how their culture is relevant to a professional encounter. *Cultural intelligence* is the most recent derivative of cross-cultural education and has gained prominence in business management settings [23]. It is a multi-faceted concept encompassing the ability to recognize differences and similarities between cultures in any given situation, the capacity to adjust and cope in unfamiliar cultural contexts, to enhance interest in learning about other cultural groups and the ability to plan and employ these skills in cross-cultural interactions. Workshop themes 2 (belief systems) and 3 (interaction approaches) are canvassed, however these themes are often presented as higher-order skillsets rather than culturally-specific phenomena.

The five cross-cultural education philosophies outlined above have on face-value, a number of potential benefits. They encourage learning about and incorporating into practice, the customs, beliefs and idiosyncrasies of multi-cultural groups, as well as querying one’s own personal biases. Anticipated outcomes from these processes include the fostering of tolerance and empathy and avoiding cultural transgressions and misunderstandings. Improved cross-cultural service delivery and workplace relations are the greater objectives. Despite these well-intentioned ambitions, the philosophical and practical shortcomings of cultural education concepts have been rarely scrutinized and warrant some unpacking. Drawing from clinical experience and the relevant health/mental health services literature, a critique of the trainings will be delineated followed by a review of their value in various settings.

### The critique

#### Superficial

Workshops are rarely long enough to absorb meaningful information that can be implemented into practice. While some trainings include more than 30 h of content, they are likely to be condensed across a two or three day period with little continuation beyond the initial workshop. Attendees will spend a part of a workshop participating in various ‘icebreaker’ games and symbolic interactive activities. For cultural awareness styled trainings, much of the remaining time is exhausted on trading in cultural stereotypes and surface-level information. This is often termed the ‘museum approach’, whereby attendees are briefly exposed to a catalogue of cultural artefacts and traditions [24]. It is doubtful that such information realistically characterizes the anticipated behaviours of most cultural minorities or the situational nuances typical of cross-cultural encounters. Moreover the artificial adoption of presumed idiosyncratic cultural phrases or traits for rapport development runs the risk of appearing insincere or patronizing. These limitations were in fact recognized over four decades ago, in one of the first official manualized cross-cultural training programs. The 1970 cross-cultural training guidelines for the United States Peace Corps state that ‘a premise of this [cultural awareness] model is that a person does not learn to exist effectively in another culture simply by being provided with information about that culture’ [[25] p12]. Contemporary workshops appear to have overlooked or deviated from, this incipient observation.

#### Essentialized

By definition, cross-cultural training almost has to be essentialized to justify its existence; if there are no obvious distinctions, then training is perhaps redundant. Yet the essentialized workshop is in danger of conveying an exoticized, romanticized or over-traditionalized description of a particular cultural group [26]. This may not accurately portray the living reality of many people from that group whose circumstances will undoubtedly be impacted by other social phenomena [27]. Although some awareness of culturally unique behaviours allows for a nomothetic heuristic, it offers little evidence for a given individual. In reality, attachment to culture varies widely – some may only ascribe to particular aspects of a culture, some may be attached to multiple cultures, others may have only a nominal or symbolic bond to a culture [28, 29]. Moreover, an individual’s engagement in non ethno-racial cultures or sub-cultures (i.e., professional, political, religious, sporting, sexual orientation etc.) may trump the importance of their ethno-racial culture. Ironically, historical discrimination against minority clientele in health settings occurred partly because clinicians *held* cultural stereotypes. Today’s workshop similarly (and precariously) maintains a ‘cultural lens’, despite couching the exercise in anti-prejudicial terms.

#### Cultural overshadowing

Cultural awareness training intentionally places cultural issues at the centre of colleague/client interaction irrespective of their relevance to a given situation. There are several potential consequences of employing a ‘cultural first’ mentality. First, any behaviour or misunderstanding may be perceived as culturally-oriented when it may not. An adverse cross-cultural interaction may be induced by a whole host of interpersonal or environmental matters that similarly impact intra-cultural encounters. Holding pre-conceived notions of an individual’s behaviour based on their cultural background may also lead clinicians to ascertain various (problem) behaviours or personal idiosyncrasies as culturally normative when they may reflect genuine psychopathology and the need for treatment [30]. Second, a focus on culture needlessly diverts attention away from potentially more significant intersecting influences on attitudes, behaviours and communication styles (i.e., age, gender, class, level of education, language proficiency, cognitive disability, personality type, psychological health, etc.). Practitioners may be less inclined to be supportive or provide the full range of treatment services to patients from lower socio-economic status backgrounds, patients deemed to have a lower health literacy, patients who have previously not adhered to treatment, patients with insufficient health care coverage, or patients with serious mental illnesses or are drug users [31]. At times, clinician unresponsiveness may be prompted by difficult/challenging patients or the clinician’s own stress levels. These scenarios are best avoided, yet they underscore possible alternative reasons for communication breakdowns between cultural groups. There is a danger in viewing every workplace or service delivery misunderstanding as a symptom of cultural incompetence. It may be the case that poor service delivery or communication is commonplace at a particular organisation and therefore generically incompetent, rather than specifically culturally-incompetent.

#### Divisive

By highlighting inter-cultural distinctions, cultural awareness training effectively creates an in-group and an out-group. This might be conveyed in multiple ways: i.e., members of a specific cultural group as distinct from everyone else; minority patients as distinct from majority culture clinicians etc. The undefined outgroup (i.e., the intended target of the workshop) is indirectly (sometimes directly) understood to be Western majority culture or perhaps more pointedly, the White Anglo male. Workshops that accentuate notions of White privilege, structural oppression and power imbalances reinforce this binary [32]. Moreover, instructors occasionally deliver training in a vindictive way, invoking far-reaching social statements (i.e., workplaces are institutionally racist or extensions of colonization; majority culture clinicians are innately privileged and have racial blind-spots). This ‘shame and blame’ approach unsurprisingly induces resentment and backlash from some workshop attendees who perceive the central message to be accusatory and that they are collectively at fault for cross-cultural predicaments. Many attendees may already feel somewhat patronized as they are obligated to attend a workshop that by definition implies that they require guidance on ‘how not to be prejudiced’. The divisive workshop rhetoric can also backfire by increasing bias or by demotivating attendees to commit to improved cross-cultural strategies [33, 34]. In some cases, attendees may subsequently feel reluctant to engage naturally with particular cultural groups, preferring to avoid contact or use prosaic and excessively cautious language [35]. At the extreme end, some may decide to ‘de-professionalize’ in order to avoid perceived power balances or curtail the impact of their intrinsic privilege of which they are taught, might be oppressing their colleagues and clients of colour [32]. The divisive framing of cross-cultural interactions as essentially ‘culture-clashes’ that require behavioural adjustment from the ‘out-group’ only, appears to indulge our natural impulse for in-group favouritism and ensuing out-group derogation [36]. This may only serve to segregate, heightening unresponsiveness or even animosity towards cultural distinctions, but most of all failing to secure the necessary ‘buy-in’ from workshop attendees. As described above, attendees relegated to the ‘culturally unaware’ outgroup may respond with indifference, antagonism, or for those feeling especially paralysed by history, choosing to surrender elements of their expertise. Neither outcome benefits cross-cultural relations.

#### Infantilizing

Workshops are laden with bleak information about the collective struggles, health discrepancies, historical injustices and suffering endured by the designated cultural group. While this information may provide an indirect backdrop to current disadvantages it does little to help attendees improve their capacity to communicate more effectively with different cultural groups. Worse, such information may only evoke pity, and for some consolidate a view of helplessness or a downtrodden stereotype [37]. This negative portrayal of members from a particular cultural group denies those members personal agency, rendering their survival or personal decision making as entirely subordinate to the abstract vagaries of society, or to the nature of workplace relations and conditions, or even to a group of well-meaning professionals attending a cross-cultural workshop. It is doubtful that the bulk of cultural minorities expect colleagues or health professionals to be entirely well-versed in, or understand their obscure cultural history/background and for those colleagues/professionals to then automatically employ an interactive style reminiscent of this cultural tradition. Migrants anticipate their local associates to have different customs and will often themselves, willingly make the necessary adjustments as they integrate. Locals who over-conform to the cultures of newly arrived migrants may be viewed as insincere.

An unattainable standard arises from describing an entire cultural group as perpetually traumatized, grieving or vulnerable – that is that such a group should expect comfort in all cross-cultural scenarios [38]. And further, if comfort is not sustained, then some form of discrimination must have transpired. This state of affairs is not possible in the course of human interaction which is loaded with miscommunications, faux pas, embarrassment, conflict and feelings of being judged. In mental and general health-care settings, patient discomfort will accompany any intrusive procedure. The idea that cross-cultural communication cannot be enhanced until all workplaces are purged of any vestige of bias and the comfort of protected groups is preserved, is unrealistic and misguided. Moreover, not all cross-cultural communication breakdowns are damaging [39]. Many are positive learning moments as individuals navigate their way through complex, dynamic environments.

#### Impractical

Professional development training is often critiqued for its limitations as an applied exercise and capacity to effect meaningful change [40]. Alone, a finite workshop is unlikely to change behaviour or an institutional culture. Clinicians who practice in demanding, high-pressure and time-poor clinical settings (especially settings with regular exposure to human suffering) are acutely aware of the constraints these environments have on skills gleaned from short professional development exercises. Moreover, some practitioners will have developed ‘empathy-burnout’ from practicing in such environments, diminishing their enthusiasm for cultural training. For attendees who are genuinely enlightened by their cross-cultural workshop experience, many will fail to recall, or struggle to implement the knowledge. This is almost inevitable if they work in a system that does not (or does not know how to) support or supervise the administering of the new knowledge [41, 42]. Moreover, workshops for health professionals will often attempt to convey nebulous cultural concepts (i.e., holistic models of health, spirituality and meta-physics, connection to nature). Apart from being difficult to operationalize (and at times fetishized), such cultural esoterica almost certainly cannot be explained in the duration of a workshop, leaving no prospect of being utilized meaningfully in practice. Mechanisms to combat cross-cultural challenges are also vaguely determined in the cultural awareness literature. Self-reflexivity, often touted as an antidote to personal bias is an entirely subjective exercise with no objective quantification [43]. This inward-looking exercise also presents an obvious conundrum – that is, the circular introspective rabbit-hole that proceeds a biased individual trying to identify their own biases. Ideally, health professionals should focus their full attention on the clinical task at hand, rather than distracting themselves with arbitrary self-corrective exercises. Furthermore, self-reflexivity is pessimistically framed as an activity whereby participants are required to locate and dwell on their own deficiencies. This stationary inward-looking exercise necessitates some reification and evidence of sustained behavioural change to support its bias reduction claims. Efforts to diminish power imbalances also suffer from unclear direction. The assumption that all majority-minority workplace interactions or hierarchical situations constitute problematic power differentials is over-generalizing and unconstructive. Professionally, power imbalances are expected and are often associated with an advanced skill set and higher levels of authority and responsibility. For example in medical settings, a complete deferral to the clinician and their expertise often trumps rapport development and informality for many minority patients from ‘rank conscious’ or traditionally hierarchical cultures. Here, a clinician who attempts to flatten hierarchy in order to ingratiate themselves with their (underprivileged) minority patient, may be viewed as amateurish or incompetent. Moreover, cultural minority status should not be considered synonymous with disempowerment. This only serves to entrench the so-called cultural power imbalances the cultural awareness industry is attempting to dismantle.

The evident impracticality of a short-lived workshop on individual and organisational attitudes and behaviours suggests that there are broader financial, political and socio-historical objectives to running the training. A clear motive is for the organisation to give the impression that diversity matters are of importance to them. While this may be genuine in some cases, cultural awareness training often exists symbolically as a corporate ‘tick-box’, or perhaps even as a protection against litigation [33, 44, 45]. One view is that organisations adopt diversity training mechanisms for ‘ceremonial’ purposes’, with full knowledge of the weak evidence base [46]. This is to give the appearance of organizational legitimacy and alignment with contemporary social movements. Others proffer that diversity initiatives are facilitated by genuine advocates, who ignore or discount the evidence in their zeal for change [46]. For many workshop instructors, there is a strong financial incentive to persist with workshops that have little long-term utility, given the widespread demand for perfunctory training. Some workshops purely exist as an exercise in social justice, described earlier as ‘vindictive’ in nature. Here, the training is less about practicality and more about stoking guilt and the enforced acknowledgement and rectification of past injustices. These workshops have a tendency to over-politicize thorny workplace relations which in reality, are mostly too mundane to have been spawned from an under-appreciation of historical inequities. Moreover, routine clinical encounters are often not multi-faceted or capacious enough to accommodate vexed broader socio-historical issues. Conventionally, the specific aim of a clinical interaction is to address an immediate medical concern, not address history. Although one may be conscious that the latter might circuitously affect the former, organizational feasibility and resource realities will preclude such grand-scale remedial action.

### The evidence

Several systematic reviews and meta-analyses have appraised the effectiveness of cross-cultural training interventions in health care environments over the past 30 years [2, 10, 12, 47–54]. The evidence can be crudely reduced to four key themes. First, there is evidence to suggest that cross-cultural training can improve the knowledge, confidence and attitudes of health professionals, albeit temporarily post-intervention. Second, there is some evidence that patient satisfaction with the clinical encounter improves after health professionals undergo cross-cultural training. Third, evidence for improved patient-related outcomes is decidedly weak. Fourth, the methodological rigor of cross-cultural intervention evaluation research is considerably poor. While some reviews attempt to put a positive spin on these findings, the extant evidence base appears to be unfavourable – particularly in regards to patient outcomes. Evidence for the impact of anti-prejudice or bias reduction interventions is equally weak [41]. Reviews and meta-analyses of both experimental and field studies (in health and non-health related disciplines) have demonstrated that changes in implicit bias (if occurring) did not translate into behavioural change [53, 55, 56]. In fact it has been found that even when physicians hold implicit racial biases, their cross-cultural clinical decision making is not necessarily impacted [57]. Moreover, the evidence for health care professional racial bias on patient outcomes is unclear [58, 59].

Evidence for the efficacy of cross-cultural/diversity initiatives in the corporate domain is also, at best, equivocal [33]. Few programs demonstrate significant value, and of the programs that do, most are developed without diversity necessarily in mind. A further concern is that the bulk of corporate cross-cultural training initiatives are laced with negative messaging which can result in counterproductive outcomes (for e.g., employee animosity; fewer minorities in management positions) [33, 46]. A study investigating which prejudice reduction strategies decrease prejudice discovered that initiatives perceived to employ control or shame and blame tactics actually increase prejudice [34]. Given that cultural awareness training was developed with specific outcomes in mind (i.e., reducing inequalities, meeting patient needs, and increasing diversity) it must be said, that the training has unequivocally failed in achieving these ideals. The literature is clear - cultural awareness training and its derivatives, (let alone its weakest format - the workshop) appear to exist almost entirely on face value. It is hard to imagine another initiative, particularly in health settings, that has persisted as ubiquitously (and often mandatorily) with unrivalled administrative support and resources for numerous decades without any robust confirmation of its practicality.

### The future

Culture awareness workshops will linger into in the foreseeable future. Based on the evidence alone, they should indeed be abandoned. Yet they will continue to be facilitated by organizations (well-intentioned or otherwise) who are keen to demonstrate their commitment to social justice causes. Greater efforts to bring attention to the failures of cultural awareness training are needed. Perhaps this may prompt the development of a ‘what works’ or ‘best practice’ literature, as opposed to a ‘well-meaning’ literature. This must begin with an honest appraisal of the question – ‘how do we improve effective cross-cultural communication?’ Before diving into cultural tropes and activism, perhaps the first response should be *– how much, if it all does one need to know about an individual’s cultural background in order to treat or work effectively with them?* I discussed earlier the pitfalls of overweighing a factor based on cultural demographics. A focus on culture often leads to essentialism. It may first be beneficial to collect localized data on how many minority patients in a designated catchment area are presenting clinically in culturally unique ways – what are the base rates of culturally-bound syndromes? It may be that most cultural minorities in a particular region are ‘mainstream’ and indistinguishable from the general population which means that cross-cultural workshops, commonly framed from an ‘outlier’ perspective are irrelevant. Moreover, this distortion is misleading and ignores the various (and more relevant) reasons why a professional encounter or relationship may break down. To fully understand why this occurs, culture must be considered like any other factor - without a predetermined emphasis. Future studies could be conducted with multi-cultural patients to identify the underpinnings of negative perceptions of health service delivery. It is important to delineate whether (or how much) negative perceptions are prompted by mistrust (real or imagined), health service provider discrimination (real or imagined) or other communication barriers (clinician apathy; patient non-compliance, transference/countertransference) and the extent to which these feelings are stimulated by personal/family experiences, historical/pre-migratory experiences or a function of the individual’s psychological profile (i.e., neurotic, hyper-vigilant, given to hostile attribution bias, cognitive difficulties). Any gripes must be compared with majority culture patients who may share similar grievances. As stated earlier, poor cross-cultural communication may be simply universally poor communication. Outside of providing translator/interpreter services (and improved linkage with same-language health professionals, [60]), interventions to enhance communication may be better aimed generically as opposed to select cultural groups which heightens essentialism.

If cross-cultural differences are found to impact the course of a clinical encounter, then other potential corrective approaches should be considered beyond being well versed in historical events or pre-emptively memorizing a list of cultural stereotypes. Cultural workshops currently exist as a deficit model – as in their content was originally derived from instances/complaints of poor practice [39]. This is why workshop content mainly involves the compelled recognition of past injustices. Workshop content was not spawned from rigorous empirical observation of effective cross-cultural practice. Perhaps this is a future direction for research – what defines effective patient/provider communication? It may be that possessing generic clinical traits such as open-ness, flexibility, customer service, listening skills and compassion induce positive experiences cross-culturally as opposed to cultural knowledge [61]. Again, the feasibility of any approach designed to alter clinical attitudes and behaviours will depend on the latitude workplace settings afford. Modifying the increasingly managerial climate in clinical settings - where efficiency and budgetary concerns overburden clinicians and restrict the capacity for longer nuanced assessments – may be necessary before realizing (or introducing) efforts to improve patient/practitioner experiences. Such settings often leave patients feeling rushed and with little time for question asking or additional assistance. It is likely that these episodes disproportionately occur among patients who are perceived by clinicians to have little interest, involvement or understanding of their own medical care or are serial non-adherents to treatment. A sensitive cultural minority patient may perceive a crude assessment conducted under these conditions as akin to mistreatment on the basis of their cultural background. Moreover, clinical biases are more likely to manifest when clinicians are stressed, tired and overworked. As such, general changes in broader workplace protocols and habits may improve the clinical encounter at large, and by extension, cross-cultural communication [33, 45].

The encumbrances cultural awareness training inflicts on clinicians are often unreasonable. First, clinical care has been shown to have only a small impact on patient outcomes [15] and on improving population health at large [62]. Second, laying the blame for cultural inequities at the feet of clinicians is misguided. A lack of cultural knowledge does not immediately signal that a clinician is disinterested, incapable or less committed to providing suitable care for a minority patient. Finger pointing will produce inadequate clinicians, some of whom may ‘soften’ their clinical approach to minorities. Some may avoid making difficult clinical decisions (that they would ordinarily make for mainstream clients) to avoid any possible perception of racism [35]. Others may plunge into confusion when they are told that their objective perceptions of challenging or complex clients, are prejudiced. For example, a recent study found that emergency department clinicians in the Australian Northern Territory perceived their Indigenous patients to have more complex health problems and that these health concerns were largely because of the patient’s lifestyles [63]. These beliefs remained unchanged despite undergoing Indigenous cultural awareness training (which presumably was expected to alter these perceptions). If emergency staff are frequency confronted with Indigenous patients who present with complex health concerns as a result of dysfunctional living circumstances, then why would their perceptions necessarily change, or be derided as culturally incompetent? The focus on clinician deficits appear one-sided. As the clinical encounter comprises both patient and clinician, negative cross-cultural interactions might at times, be patient-induced. In some communities, there exist unhelpful narratives proclaiming that all ‘outgroup’ clinicians are racist and do not always operate in the minority patients’ best interest. Such attitudes may have some historical truth and/or reflect a contemporary isolated incident, yet are harmful when widely and determinedly shared with community members. This works against clinicians who are already saddled with cultural training messaging eagerly informing them of their unbeknownst in-built prejudice towards underprivileged cultural groups. A re-think of clinician-focused initiatives is warranted, and if some derivative of cultural awareness training endures, then community outreach attempts should also be included to ameliorate negative community attitudes.

For individuals working in foreign environments, a basic understanding of cultural norms may be necessary, depending on the extent to which the new environment differs from the individual’s home culture. However, preliminary information should be restricted to specific local customs, which if transgressed, could put the clinician or their patient in harm’s way, or preclude the clinician from capably or safely administering care. Minor cultural differences will be negotiated as a clinician acclimates to their new surroundings. Naturally, individual patients, even in unfamiliar homogenous locations will possess myriad concerns and present idiosyncratically.

Last, the relevance of a patient’s culture to a clinical assessment can be established and verified without having pre-conceived expectations. One worthwhile aspect of the cultural humility approach is to allow the patient to determine how much bearing their culture has on their health concern. For mental health clinicians the DSM Cultural Formulation Interview includes prompts for clinicians that may elicit answers to this question [64]. Some of these supplementary questions may assist clinicians in identifying cultural perceptions of illness (i.e., how would you describe your issue to your community?; What does your family/community think is causing your problem?; Why did it start when it did?). Again, the clinician should interpret such responses in the broader context of a patients unique positioning across multiple sub-cultures and other environmental factors without fixating on culture alone.

## Conclusions

Cultural awareness workshops and their derivatives are often well-intentioned and genuine efforts to improve cross-cultural engagement in health care settings are a laudable pursuit. Yet these interventions are implemented without evidence and exist on face validity alone. Decades of research point to their ineffectiveness, despite billions of dollars being spent on their operation. Workshop approaches are often over-generalizing, simplistic and impractical. Broader expectations of reductions in health disparities are almost certainly unachievable. It is right to have high expectations for health care practice and clinician performance. But we must also consider that clinical assessment (and human interaction at large) is often a woolly fact-finding endeavour involving trial and error, the generation of numerous mini-hypotheses, pragmatism and micro-decision making, undertaken in an imperfect and sometimes frenzied organizational context. The complexity of interaction and behaviour cannot be reduced to facile insider-outsider, majority-minority, privileged-underprivileged narratives. The cross-cultural workshop should be retired until there is sufficient evidence for its necessity, let alone its utility.
